# Mimicking Transmural Helical Cardiomyofibre Orientation Using Bouligand-like Pore Structures in Ice-Templated Collagen Scaffolds

**DOI:** 10.3390/polym15224420

**Published:** 2023-11-16

**Authors:** Huijie L. Zhang, Sanjay Sinha, Ruth E. Cameron, Serena M. Best

**Affiliations:** 1 Department of Materials Science and Metallurgy, University of Cambridge, Cambridge CB3 0FS, UK; 2 Wellcome Trust-MRC Stem Cell Institute, Department of Medicine, University of Cambridge, Cambridge CB2 0AW, UK

**Keywords:** freeze drying, cardiac tissue engineering, polymer processing, anisotropic porosity

## Abstract

The helical arrangement of cardiac muscle fibres underpins the contractile properties of the heart chamber. Across the heart wall, the helical angle of the aligned fibres changes gradually across the range of 90–180°. It is essential to recreate this structural hierarchy in vitro for developing functional artificial tissue. Ice templating can achieve single-oriented pore alignment via unidirectional ice solidification with a flat base mould design. We hypothesise that the orientation of aligned pores can be controlled simply via base topography, and we propose a scalable base design to recapitulate the transmural fibre orientation. We have utilised finite element simulations for rapid testing of base designs, followed by experimental confirmation of the Bouligand-like orientation. X-ray microtomography of experimental samples showed a gradual shift of 106 ± 10°, with the flexibility to tailor pore size and spatial helical angle distribution for personalised medicine.

## 1. Introduction

**Figure 1:**
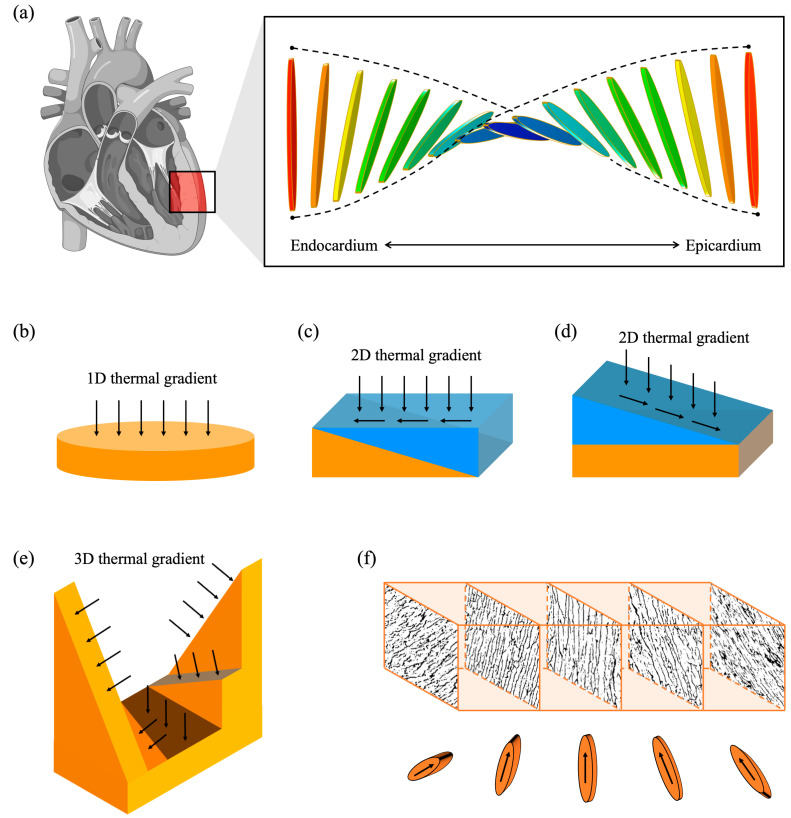
The base design in ice templating. (**a**) Overview of the cardiomyofibre orientation in native heart. (**b**–**d**) Base designs in ice-templating method to introduce 1D and 2D thermal gradients for freezing the slurry, where orange and blue represent different thermal conductivity. (**e**) Our Bouligand-like orientation design introduces 3D thermal gradient and (**f**) the experimental confirmation for the base design.

The muscle fibre orientation across the heart wall is a highly complex three-dimensional (3D) arrangement, which constantly changes throughout the cardiac cycle (diastole and systole). The muscle fibres form layers that wrap helically around the heart chamber, and the helix angles in the layers vary gradually across the heart wall. As shown in Figure 1a, inside the wall of the left ventricle, the fibre orientation changes from a left-handed helix to a right-handed helix from the epicardium to the endocardium. A wide range of helical angles has been reported across the wall, from 90° to 180°, depending on species and detection methods [1,2,3,4]. This transmural cardiomyocyte orientation governs the mechanical and electrical properties and thereby the cardiac function. It accounts for the overall twisting motion of the chamber [5], systolic wall thickening [3,6], and achieving large ejection fractions [7,8]. It is crucial to replicate this complex architecture to develop mature and functional in vitro models.

For artificial cardiac constructs, replicating such 3D architectures in tissue engineering scaffolds remains a problem. Local alignment on the cellular level has been achieved using a variety of methods, including electrospinning [9,10,11], freeze-drying [12,13,14,15,16,17,18], 3D printing [19,20,21,22,23,24], and rotary jet spinning [25]. This architectural feature in tissue-engineered scaffolds was proven to guide cell alignment and improve cell maturation [9,17,26,27]. However, previous researchers have focused on a single-oriented alignment for cardiac patches, essentially constructs with only two-dimensional architectural features, and very few have successfully reached the level of complexity in the heart. The fabrication methods for such structures have been relatively limited except for direct processing from the natural organs (e.g., decellularised extracellular matrix [28,29]). There are some bottom-up techniques available that shed some light on this matter. One method involves stacking aligned cardiac patches into a layered 3D construct with offset angles designed to resemble the orientation shift across the heart wall [26,30,31], yet the ways of creating the single-alignment pattern in one individual layer can vary. Fleischer et al. combined laser patterning and electrospinning to achieve the alignment [31], whereas Wu et al. proposed weaving nanofibre yarn to guide cell orientation [26]. It is also possible to create patterned cell layers without conventional scaffolds. Takahashi et al. functionalised the surface of the cell culture dish to obtain myoblast sheets with inherent cell orientation [30]. Overall, the stacking approach requires the precise handling of layers with the offset angle and suffers from a lack of connection between layers. Nonetheless, cell culture in thin layers may be preferred due to the nutritional diffusion limit, especially when compared with culturing cells in large constructs, which requires an appropriate seeding protocol or perfusion system to ensure cell survival. On the other hand, fibre spinning and 3D printing can build 3D continuous networks with better layer-to-layer connections [8,20,24]. Yang et al. used electrically assisted 3D printing to take advantage of the Bouligand structures to improve the mechanical properties of artificial human meniscus [20]. This system had a much higher modulus compared to the cardiac tissue but still works as a potential way to solve the problem. Furthermore, the recent work of Choi et al. utilised 3D printing to create a single helical layer of the heart chamber [24]. Chang et al. successfully developed a rotary jet printing technique to mimic the helical alignment within the whole heart [8]. Although this showed potential in recreating the complex architectures in the heart chamber, the 3D structural definitions within the network still rely on fibres overlapping with each other due to the very nature of these techniques. A different approach is therefore needed to fabricate scaffolds with a continuous 3D network.

One of the benefits of ice templating is its simplicity and the potential to fabricate architectures with interconnected pore networks, which is pivotal to 3D cell culture. Cells can recognise anisotropic pore structures and show cytoskeletal alignment with the orientation of pore walls [17,27]; hence, well-defined spatial structural anisotropy in the pore structures can lead to cell organisation in artificial tissue, potentially recreating the native structural hierarchy in vitro. However, to date, the technique has not been utilised to mimic the transmural arrangement of cardiomyocytes due to the difficulty of fabricating such complex structures. There are three stages in the ice-templating process: freezing, primary drying and secondary drying [32]. The freezing stage is critical in determining the pore features [33]. The key to obtaining aligned porosity is unidirectional ice solidification, which can be achieved by combining a highly conductive metal base and relatively insulating walls into a mould [13,18,34,35]. We hypothesise that by simply controlling the topology of the base, we can influence the thermal profile during the freezing stage and thereby determine the final pore structure. Many researchers used a flat base that was made of a single material to produce aligned pore structures, and the corresponding pore orientation is noted as 1D in this paper (Figure 1b). If multiple materials were used (Figure 1c), the flat base is then capable of introducing 2D thermal gradients, therefore influencing the ice growth direction [13,36]. Wedges can also introduce dual-directional freezing with a tilting base, as demonstrated in Figure 1d. Bai et al. and Zhang et al. placed a polydimethylsiloxane (PDMS) wedge on a copper sheet, producing lamellar structures [37,38]. Additionally, radial and radial-concentric freezing rely on the 1D thermal gradient but on a curved surface, leading to a 2D alignment orientation [39]. Other objects can also be added to the slurry to introduce a customised heat flow [40]. Furthermore, instead of designing the freezing system based on trial and error, heat transfer simulations can be used to predict the thermal gradient, hence the final orientation of the aligned pores [18,40,41,42,43]. This accelerates the mould design for ice templating and helps to unleash its potential with more complicated thermal gradients.

Here, we describe how we successfully recreated the complex helical architectures across the heart wall in ice-templated collagen scaffolds via multi-directional freezing. We utilised finite element modelling as a rapid design tool to tailor the 3D thermal gradient to induce ice growth to match the helical angle variation across the heart wall. We then propose an experimental design with a 5-wedge base, as shown in Figure 1e, which successfully fabricated the above complex architectures in collagen scaffolds (Figure 1f) with a smooth transition in pore orientation. With the analogy to a twisted plywood structure or Bouligand structure, we named our base design a Bouligand-like orientation design.

## 2. Materials and Methods

### 2.1. Finite Element Simulation

The heat transfer during the freezing stage of ice-templating was simulated in COMSOL Multiphysics 5.6^®^. Two mould designs were investigated to study the influence of base geometry: the wedge base design and the Bouligand-like orientation design. Both moulds were constructed using a copper base and a polycarbonate body, and the freezing of the slurry was simulated by water/ice phase change. Table S1 records all essential thermal parameters for the simulation. The 3D heat diffusion equation (Equation (1)) was used to calculate the temperature at any given time during the simulation, where T is temperature, t is time, ρ is density, $C_{p}$ is specific heat capacity, k is thermal conductivity, and Q is the sum of heat.

(1) $$\rho C_{p}\frac{\partial T}{\partial t}+\nabla (-k\nabla T)=Q$$

The phase change between water (w) and ice (i) was simulated using a transitional mushy zone, which was defined as a temperature zone over a 4 °C interval centred at 0 °C. The phase proportion (θ) followed a smoothed step function between 0 and 1 in the temperature zone. Therefore, the water phase proportion ($\theta_{w}$) and the ice phase proportion ($\theta_{i}$) in the mushy zone had a sum of 1, as shown in Equation (2).

(2) $$\theta_{w}+\theta_{i}=1$$

The density, thermal conductivity, and specific heat capacity of the transition zone can then be described using Equations (3)–(5), where $L_{i\to w}$ is the latent heat (333.5 kJ/kg).

(3) $$\rho =\rho_{w}\theta_{w}+\rho_{i}\theta_{i}$$

(4) $$k=k_{w}\theta_{w}+k_{i}\theta_{i}$$

(5) $$C_{p}=\frac{1}{\rho }\left({\rho_{w}\theta }_{w}C_{p,w}+{\rho_{i}\theta }_{i}C_{p,i}\right)+L_{i\to w}\frac{\partial }{\partial T}(\frac{1}{2}\frac{{{{\rho_{w}\theta }_{w}-\rho }_{i}\theta }_{i}}{{{{\rho_{i}\theta }_{i}+\rho }_{w}\theta }_{w}})$$

Three boundary conditions were imposed during the simulations: the initial temperature, heat extraction at the mould bottom, and heat exchange with the surrounding environment. The initial temperature was set to be 20 °C for all domains. The heat extraction at the mould bottom was set to be in line with a freezing protocol. For both the wedge base mould and the Bouligand-like orientation mould designs, the temperature of the bottom surface of the base (the contacting surface between the mould and the cold finger) was set as −10 °C for 1 min of stabilisation at the start of the simulation, followed by continuous cooling at the rate of 1 °C/min. Finally, the heat exchange with the surrounding environment was simulated using convective heat flux. Equation (6) was used to calculate the heat flux, where $h_{c}$ is the convective coefficient, $T_{ext}$ is the environment temperature, and $T_{surf}$ is the surface temperature of the mould. $h_{c}$ was set as 27.5 W/(m^2^K) for all simulations [18,43].

(6) $$q_{c}=h_{c}(T_{ext}-T_{surf})$$

### 2.2. Scaffold Fabrication

1 wt% insoluble bovine dermal collagen powder was hydrated in 0.05 M acetic acid overnight. The collagen suspension was then blended and degassed under vacuum to form a homogeneous slurry. The slurry was then poured into the mould under study. The mould containing the slurry was placed on a copper finger of lab-built freezing equipment [18]. This custom freezing equipment utilised liquid nitrogen to allow faster cooling rates and a wider selection range of temperatures. The cold finger temperature was regulated using proportional–integral–derivative controllers. The freezing protocol for the prototype mould was to hold the temperature of the cold finger at −10 °C for 1 min for stabilisation, and then to continue cooling at the rate of 1 °C/min until the collagen slurry was completely frozen. There were two freezing protocols used for the refined mould,; one was to hold the temperature of the cold finger at −5°C and the other was to hold the temperature at −10 °C. Once the slurry was completely frozen, the mould was transferred to a commercial freeze drier (VirTis Advantage Pro, Biopharma Group, Winchester, UK) for ice sublimation. The freeze drier was pre-cooled to −20 °C before the samples were transferred from the cold finger. Once the samples were in the freeze drier, the shelf temperature was ramped to −20 °C and then held at that temperature for one hour. The drying stage was carried out at 80 mTorr, 0 °C. The drying time for the prototype mould and the refined mould was 6000 min and 1500 min, respectively.

### 2.3. Structural Characterisation

The scaffolds that were freeze-dried using the prototype mould were cut into a prism volume of interest, as suggested in Figure S1, and then imaged using X-ray microtomography (μCT) (Skyscan 1272, Bruker, Kontich, Belgium). The X-ray source was set as 25 kV and 138 μA without any filter. The imaging pixel size was 6 μm, with the rotation step of 0.1°. On the other hand, the scaffolds freeze-dried using the refined mould were small enough for μCT scanning without cutting. The X-ray source was again set as 25 kV and 138 μA without any filter. The imaging pixel size was 4 μm, with the rotation step of 0.1°. The reconstruction of the 3D architecture was carried out using μCT images in NRecon (version 1.7.3.0), and subsequent reconstructed images were analysed using DataViewer (version 1.5.2.4) and Fiji (ImageJ version 1.54f). The stacked reconstructed images on x-z planes along the *y*-axis were evenly divided into 5 substacks, and then the directionality analysis was performed on individual slices. The 3D orientation distribution in certain regions combined all slices from one substack. The directionality histogram was then normalised to a maximum intensity of 1 for comparison.

### 2.4. Statistical Analysis

N = 4 for the samples freeze-dried using the prototype mould; N = 3 for the samples freeze-dried using the refined mould for the two freezing protocols. The error shown represents the standard deviation unless otherwise stated in the text.

## 3. Results

### 3.1. The Effects of Slope Angle in a Wedge Base

**Figure 2:**
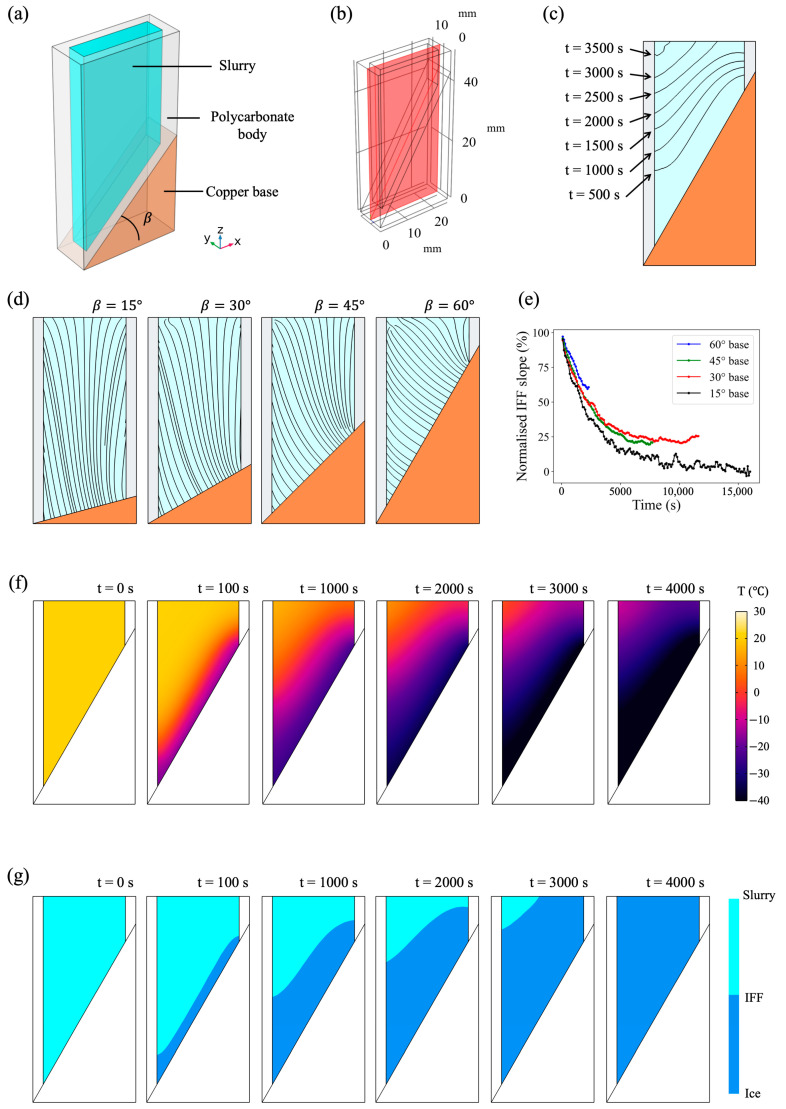
The wedge base design. (**a**) Overview of the wedge base design with illustration of β as the slope angle. (**b**) The analysis plane for orientation analysis. (**c**) The snapshot of IFF positions at 500 s intervals for the 60° wedge base mould. (**d**) When β increases from 15° to 60°, the streamlines on the analysis plane at the time of freezing completion. (**e**) The linear regression results of the IFF slope during the simulation when β changed between 15° and 60°. (**f**,**g**) The temperature profile and phase separation diagram as the simulation progressed for the 60° wedge base mould.

A mould design with a wedge base was built using COMSOL Multiphysics^®^ to investigate the effects of the slope angle (β) on the thermal gradient, as shown in Figure 2a. The wedge base was made of copper, and the insulating mould body was made of polycarbonate (3D animation of mould construction in Video S1). The temperature within the whole 3D geometry was solved over the period of 16,000 s at a time interval of 1 s. It was found that the time for the water domain to become completely frozen (temperature below −2 °C) was 16,000 s, 12,350 s, 8280 s, and 3840 s when β was, respectively, 15°, 30°, 45°, and 60°. Figure 2f gives an example of the temperature profile for the 60° wedge base mould throughout the simulation. Given the steady heat flow, the streamlines at a single time point would be sufficient to represent the preferred pore growth direction based on the thermal gradient. Figure 2d shows the streamlines based on the temperature vector field on the cross-sectional x-z plane (Figure 2b) at the completion time of the freezing stage. It was noted that the streamlines tended to be perpendicular to the tilted wedge base at the bottom of the mould but would gradually become in line with the major heat extraction direction at the top of the mould. In other words, the influences of the base geometry decreased with the distance between the current position and the base. Whether or not the streamlines at the top of the mould would finally reach vertical alignment depended hugely on the height of the mould.

To further quantify the influences of the base geometry, we used the position of the ice freezing front (IFF) at every 100 s interval throughout the simulation process. The IFF was represented by the interface of −2 °C to avoid complications of the latent heat, and this separated the domain into two phases, as suggested in Figure 2g. Figure 2c records the IFF movement in the 60° wedge base mould. The IFF slope with respect to the *x*-axis was in line with the deviation angle between the preferred pore growth direction and the vertical direction (*z*-axis). Every 100 s, linear regression was implemented to evaluate the IFF slope variation as simulations progressed. It was found that the IFF angle initially followed the base angle and decreased over time as the IFF moved towards the top. It was also noted that as IFF was near the top, the linear regression became less reliable due to the mould edges. Figure 2e summarises the normalised IFF slope variation for β was, respectively, 15°, 30°, 45°, and 60° for the wedge mould. It was found that all simulations followed a similar trend until the emergence of the edges. These results coincided with the previous qualitative streamline results that the base angle played an important role in the preferred pore growth orientation, especially near the base, and this influence became less dominating as time progressed. These results suggest that the wedge base can alter pore growth orientation, and the simulation results can be extrapolated further for general mould design.

### 3.2. Bouligand-Like Orientation Design: Proof of Principle

**Figure 3:**
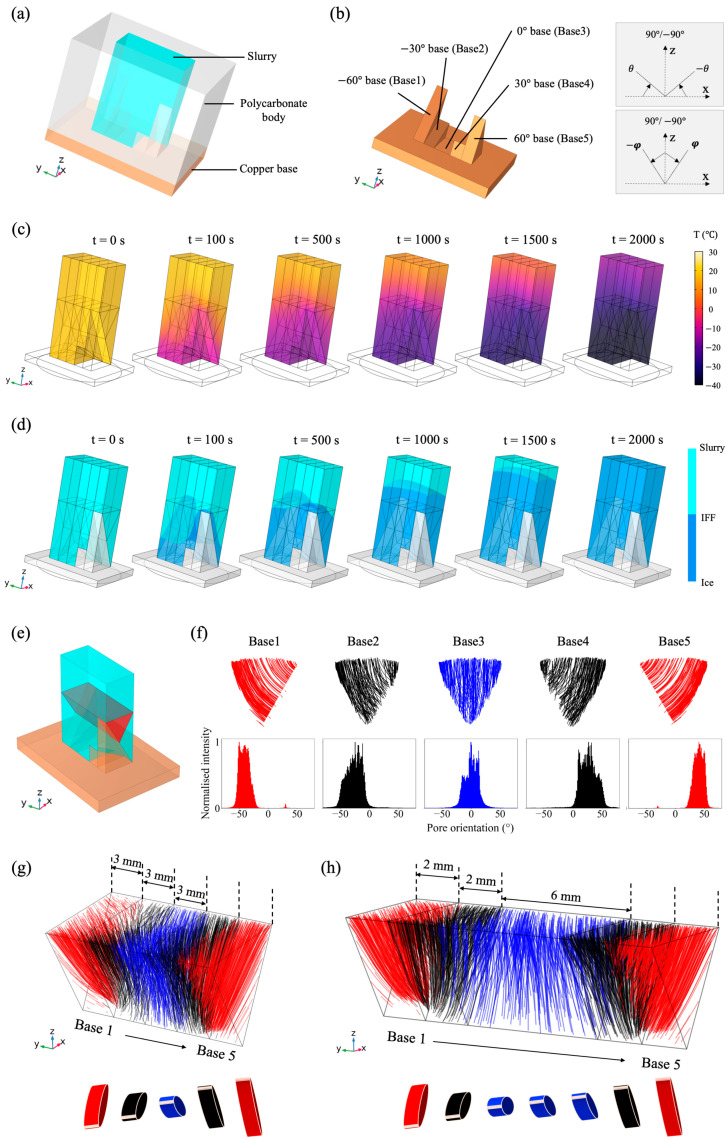
The Bouligand-like orientation design. (**a**) The overview of the Bouligand-like orientation mould. (**b**) Details of the base topography. The base angle was defined as θ, and pore orientation deviation from the vertical alignment was defined as φ. (**c**,**d**) The schematic diagram of the temperature profile and IFF movement as the simulation progressed. (**e**) The region of interest for orientation analysis was highlighted in red. (**f**) Within the region of interest, the streamlines above a certain base and its corresponding pore orientation distribution. (**g**,**h**) The 3D distribution of streamlines within the prism region for (**g**) the equal width design (prototype mould) and (**h**) the unequal width design (refined mould).

We proposed a mould design with a 5-wedge base to replicate the helical arrangement of the cardiomyofibres. Figure 3a,b show an overview of our mould design (3D animation of the mould construction in Video S2). The base angle was defined as the angle between the top surface of the wedge and the *x*-axis (denoted as θ in Figure 3b). Consequently, base 1–5 had θ values of −60°, −30°, 0°, 30° and 60°. Based on the results from the wedge base, the corresponding pore orientation was defined as the deviation from the vertical direction (*z*-axis), denoted as φ in Figure 3b. The temperature within the 3D geometry was solved at the time interval of 1 s (Figure 3c). The distortion of the IFF during the simulation was found to be the most significant near the base, as suggested in Figure 3d. Therefore, a larger variation in the pore orientation shift was expected near the base. Figure 3e highlighted a red prism region in the water domain. This region was the closest region near the base to include the influences from base 1–5 and hence was used as the region of interest for later analysis. As shown in Figure 3g, it was found that the 3D streamlines in the five regions along the *y*-axis exhibited a 3D mirror symmetry with respect to the x-z plane in the centre of the prism. Above one individual base, the 3D streamlines were flattened onto a 2D plane and orientation analysis was performed (Figure 3f). The orientation shift was identified as 82°, 68% of the wedge angle differences (120°). Furthermore, the 3D distribution of pore orientation can be fine-tuned by the width of the wedge along the *y*-axis, as demonstrated in Figure 3g,h. Overall, the simulation results suggest that the helical arrangement of pore orientation can be achieved using this bespoke mould design.

**Figure 4:**
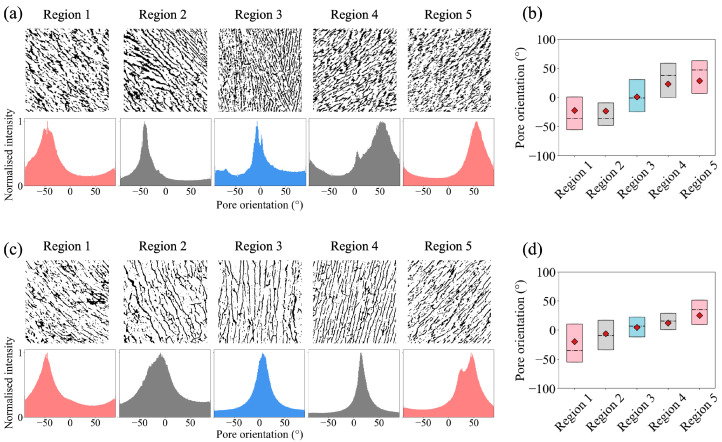
The structural analysis of the ice-templated collagen scaffolds. (**a**,**b**) The reconstructed μCT images and corresponding pore orientation distribution of the samples fabricated using the prototype moulds. The area of interest was 1.2 mm × 1.2 mm at the centre of the prism region. (**c**,**d**) The reconstructed μCT images and corresponding pore orientation distribution of the samples fabricated using the refined moulds. The area of interest was 2 mm × 2 mm at the centre of the prism region.

Three prototype moulds (Figure S1) were then made to test the idea experimentally. This mould had an overall dimension of 32 mm × 32 mm × 56 mm with 6 mm polycarbonate insulating layers and a 2 mm flat copper bottom (base 3) to hold all the other bases. 1 wt% collagen slurry was freeze-dried in the prototype moulds, and four experimental samples were fabricated in total. Figure 4a shows the reconstructed μCT images of the scaffolds within the 1.2 mm × 1.2 mm square area at the centre of the triangle area previously mentioned in Figure 3e. For the prototype mould, regions 1−5 were directly above base 1−5. The snapshot of one slice from every region shows that the aligned pores changed their orientation when the base angle changed from −60° to 60°, and the 3D directionality analysis supported this observation. Based on the peak position of the orientation histogram, an average orientation shift of 106 ±10 ° was found in four samples. Furthermore, the box plot in Figure 4b demonstrates the pore orientation distribution spread within the first and third quartiles. The large overlapping between neighbouring regions confirmed a continuous orientation change in aligned pores despite the discrete base angle design. Additionally, the change in median (dotted line) and mean values (red point) both indicate the gradual shift in the pore orientation, giving a relative 73 ± 13° and 42 ± 7° variation in the orientation shift. The peak values were used to interpret the distribution variation, while the median and mean values were more associated with the noise in the histogram background.

### 3.3. Tuneable Orientation Change for Personalised Medicine

Once the mould design was proven to be successful, we developed a scaled-down version of the prototype mould to meet clinical requirements. The refined mould design (Figure S2) had an overall dimension of 24 mm × 15 mm × 17 mm with 5 mm polycarbonate insulating layers and a 2 mm flat copper bottom (base 3) to hold all the other bases. It was noted in the prototype mould design that the equal width of all bases led to less aligned structures in the middle above base 3 because of the thermal gradient introduced by the side of the wedges. Therefore, the width of base 3 in the refined mould was also adjusted to 6 mm, while the width for the remaining bases was 2 mm. As demonstrated in Figure 3h, the simulations suggested that this unequal width design still exhibited a 3D mirror symmetry; however, this design also allowed more space for vertically aligned pores to grow above base 3. 1 wt% collagen slurry was frozen in three refined moulds at a constant temperature of −5 °C. A higher temperature was chosen here to allow a larger pore size for an easier observation. A 2 mm × 2 mm area of interest at the centre of the triangle area previously mentioned in Figure 3e was selected to monitor the changes in the pore orientation in the five evenly divided regions. Here, unlike the prototype mould, the positions of regions 1-5 were noted to differ from bases 1–5 along the *y*-axis. Figure 4c shows the reconstructed μCTimages and the corresponding orientation distribution in the five regions. A mirror symmetry was identified, and a gradual orientation shift of 83 ± 7° was found in three samples based on the peak positions on the directionality histograms. More importantly, Video S3 shows the slice-by-slice evolution of the pore structures, and a smooth transition in the pore orientation was observed. The boxplot shown in Figure 4d shows that the change was smoother when compared to the prototype mould (Figure 4b). Furthermore, this design produced a pore size in the range of 100 μm, comparable to the dimension of mature cardiomyocytes.

**Figure 5:**
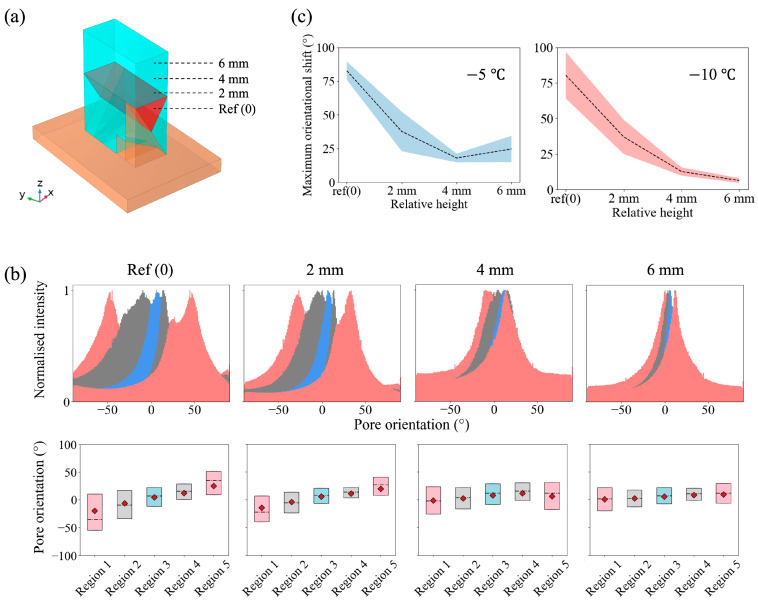
Further characterisation of the refined mould samples. (**a**) Illustration of the volume of interest position. (**b**) The pore orientation distribution when moving away from the base. (**c**) The overview of maximum orientation shift within the scaffold using two freezing protocols (holding the temperature at −5 °C and −10 °C).

It was also possible to obtain different orientation shifts within the same ice-templated scaffold. Let the region at the centre of the prism be at the reference height as 0 along the *z*-axis (h = 0 mm). Figure 5a,b shows the pore orientation distribution in the scaffold while moving further away from the bases with respect to the reference height. Based on the peak positions, it was found that the maximum orientational shift of the aligned pores decreased from 92° to 11° when h changed from 0 to 6 mm. This can be particularly powerful for further cell studies because it allows systematic studies on the scaffolds with only architectural differences in the orientation shift while keeping any other variables consistent, such as collagen batches and alignment of the collagen struts. To further assess the reproducibility, we fabricated three scaffolds with the cold finger temperature at −5 °C and −10 °C,respectively. Figure 5c shows the average orientational shift with respect to the distance to the base for both freezing protocols. As the monitoring position moved away from the base, the orientational shift became smaller. Near the base, the change in pore direction was found to be 83 ± 7° for scaffolds frozen at −5 °C and 80 ± 16° for scaffolds frozen at −10 °C. Near the top, the orientation shift was 25 ± 10° for the −5 °C group and 6 ± 2° for the −10 °C group. Additionally, lower freezing temperatures provide a larger undercooling, and the corresponding pore size was expected to be smaller. Hence, the pore size can be tuned with different freezing temperatures without compromising the pore orientation shift. It was found that the orientational shift remained similar when frozen at −5 °C and −10 °C, suggesting the flexibility of choosing pore sizes for different cell types using this technique.

## 4. Discussion

In this work, we have explored the 3D directional freezing with the aid of finite element simulations and proposed a creative solution to replicate the helical arrangement of the cardiomyofibres across the heart wall. We started with a simple wedge base mould design. The simulation results showed clear evidence that the slope angle affected the pore growth orientation, especially near the base. It was noted that the high thermal conductivity of the copper base allowed fast, almost simultaneous ice nucleation at the very surface of the base, and the geometry of the base had little effect on delaying the temperature from reaching the nucleation temperature across the entire base. Hence, a thin isotropic layer near the bottom was expected but considered negligible. Then, the ice growth was influenced by both the thermodynamics of crystal growth and the presence of mould boundaries. In this case, the combined effects led to a 2D thermal gradient that changed the initial pore orientation from the vertical direction to the direction perpendicular to the wedge base. This perspective was overseen in the previously reported work due to the choice of base materials. If the wedge was made of a poor thermal conductor, such as PDMS, then the ice nucleation would be delayed because of the horizontal thermal gradient. The combined effects would eventually lead to a lamellar structure, as demonstrated in the work of Bai et al. and Zhang et al. [37,38]. On the other hand, a similar delay in the ice nucleation was also reported in flat base design when combining different materials. Pot et al. [36] and Davidenko et al. [13] used a dual-wedge design to form a flat base surface. Two wedges have complementary shapes. One wedge was made of a good thermal conductor, and the other wedge was made of a poor thermal conductor. A horizontal thermal gradient was reported in both cases.

Then, we proposed a base design comprising five wedges to create a 3D thermal gradient with mirror symmetry that resulted in a Bouligand-like pore orientation. Similar to the wedge base design, we expected fast ice nucleation at the base surface, followed by ice growth, whose direction was heavily influenced by the thermal gradient. Both the simulation and the pilot study with the prototype mould suggested a successful pore orientation shift, confirming our hypothesis that the 3D pore orientation distribution can be controlled by the base topography via a thermal gradient. The experimental sample showed a pore orientation variation of 106 ± 10°, within the 90–180° range of the transmural helical angle change in vivo [1,2,3,4]. Our design provides a novel and easy adaptation to the well-established industrial method. It has the potential to quickly produce scaffolds in a cost-efficient way. Previous research in ice templating has tried to control pore orientation with external stimulations, such as magnetic [44] and thermal [18]. The other existing approaches to building constructs that feature helical angle variations are stacking single-orientated layers [26,30,31], 3D printing [20,24], and rotary jet printing [8]. The stacking method requires precise handling of the layers and leaves a relatively weak connection between layers, whereas 3D printing, rotary jet printing, and our proposed freeze-drying method fabricate the 3D network first. It is more likely for the latter to form a functional unity and easier to build the construct. On the other hand, both 3D printing and rotary jet printing build up the 3D hierarchy via the overlapping of fibres, while freeze-drying has the advantage of producing an interconnected network with minimal layer-by-layer disruption. Additionally, the strut bridging between vertically aligned struts often found in freeze-dried scaffolds resembles the collagen fibre bridging in the native tissue [45].

Finally, we developed a refined mould to adjust the dimension of the ice-templated collagen scaffolds to clinically relevant sizes (wall thickness: 1.4 cm) with tuneable architectural features, such as pore size, heart wall thickness, and alignment direction distribution. Our previous work has shown that it is possible to fine-tune the pore size via freezing protocol [46] and to adjust the pore wall thickness and strut diameter using different concentrations of collagen slurry [47]. Depending on age, species, and healthy conditions, the helical angle distribution across the heart wall and wall thickness can vary [1,2,3,4,48]. With the development of non-invasive in vivo imaging techniques, the specific helical angle distribution can be obtained for individual patients, and our design can be incorporated into personalised medicine. Our previous work confirmed the excellent in vitro biological performance of ice-templated collagen scaffolds using a variety of cell types [49], and this work suggests promise for the support and development of functional cardiac tissue in the future. Many questions remain to be answered in 3D cell culture, and our system offers the potential for reproducible, native, tissue-like structural environments.

In summary, we established a robust platform based on predictive modelling and experimental confirmation, successfully recreating the structural hierarchy of the native heart in ice-templated collagen scaffolds. Our computationally designed scaffolds offer a simple, scalable, and adaptable tool to build artificial constructs with customised 3D distributions of pore orientation.

## Data Availability

The data supporting this study have been deposited on Apollo (University of Cambridge Repository) at http://doi.org/10.17863/CAM.104059.

## Supplementary Materials

The following supporting information can be downloaded at https://www.mdpi.com/article/10.3390/polym15224420/s1. Figure S1: the overview of the prototype mould and sample preparation for μCT; Figure S2: the overview of the refined mould; Table S1: thermal properties of the finite element simulation; Video S1: 3D animation of the wedge design; Video S2: 3D animation of the Bouligand-like orientation design; Video S3: the reconstructed μCT images of the sample fabricated in the refined mould, showing a continuous pore orientation shift in region 1–5.
