# Supplementary material for: Mimicking Transmural Helical Cardiomyofibre Orientation Using Bouligand-like Pore Structures in Ice-Templated Collagen Scaffolds
Source: Polymers (Basel). 2023 Nov 16;15(22):4420. doi: 10.3390/polym15224420 (PMC10675392; doi:10.3390/polym15224420)
Supplement: Supplementary file 1 [file polymers-15-04420-s001.zip › supplementary_materials.pdf]

Supplementary Materials for

Mimicking transmural helical cardiomyofibre orientation using Bouligand-like pore structures in ice-templated collagen scaffolds

Huijie Zhang *et al.*

*Corresponding author. Email: rec11@cam.ac.uk (REC); smb51@cam.ac.uk (SMB)

This PDF file includes:

Figs. S1 to S2
Table S1
Videos S1 to S3

Other Supplementary Materials for this manuscript include the following:
Videos S1 to S3

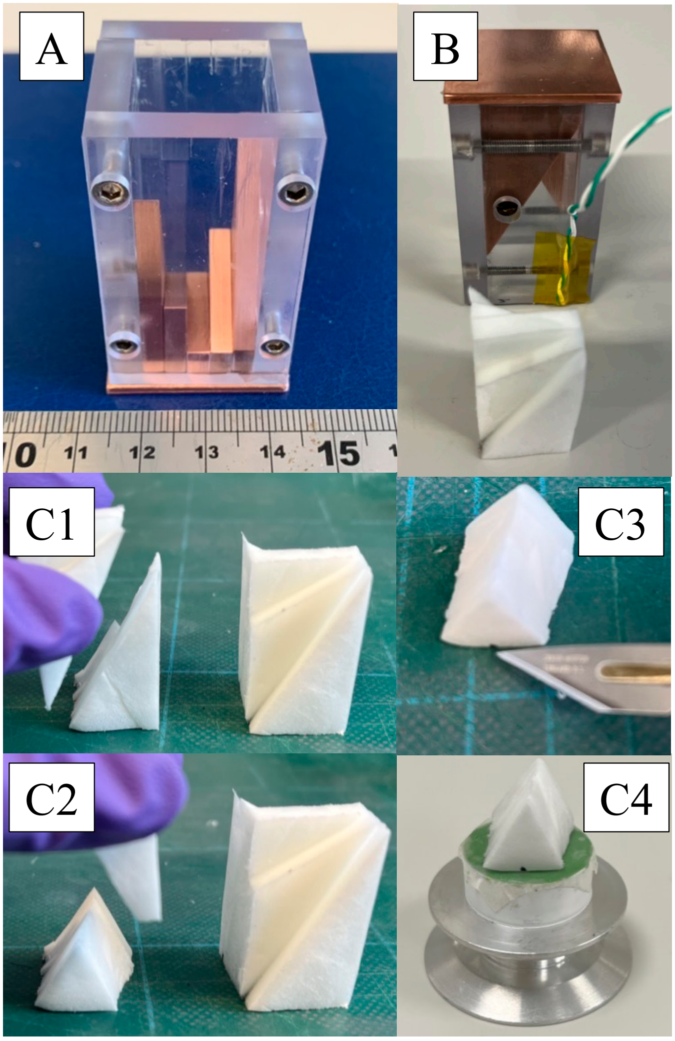

Fig. S1. The overview of the prototype mould and sample preparation for X-ray microtomography (μCT).
(A) The prototype mould. **(B)** The freeze-dried scaffold in dry state just taken out of the mould. **(C)** Detailed steps to cut the sample into the prism volume of interest for μCT imaging. **(C1-2)** Cutting of excess collagen near the base on the left hand side with the uncut sample on the right hand side. **(C3)** A black dot was to mark the prism positioning. **(C4)** The sample was mounted with double-sided tape onto the sample stage for μCT imaging.

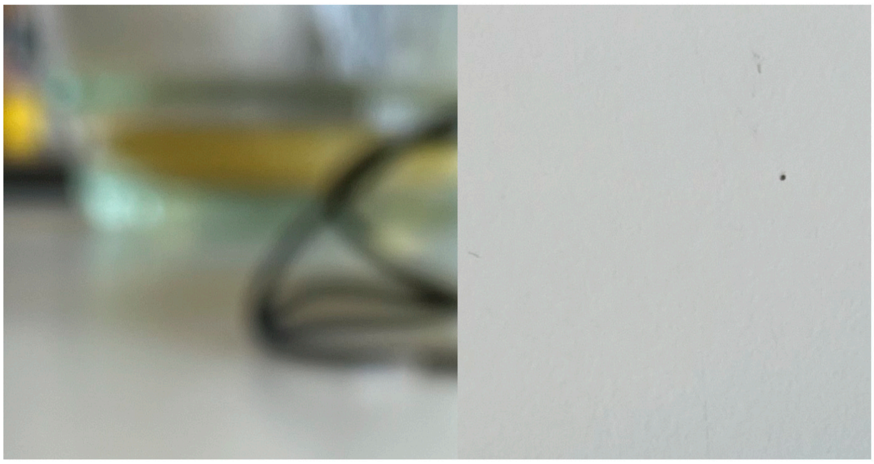

Fig. S2. The overview of the refined mould.

Table S1.
Thermal properties of the finite element simulation.

Parameter	Value	Description	Temperature-dependency
k_w	$0.6\ W\ m^{-1}\ K^{-1}$	Thermal conductivity of water	No
k_{Cu}	$401\ W\ m^{-1}\ K^{-1}$	Thermal conductivity of copper	No
k_p	$0.2\ W\ m^{-1}\ K^{-1}$	Thermal conductivity of polycarbonate	No
k_i	$2.2 - 0.05\boldsymbol{T}\ W\ m^{-1}\ K^{-1}$	Thermal conductivity of ice	Yes
cp_w	$4200\ J\ kg^{-1}K^{-1}$	Specific heat capacity of water	No
cp_{Cu}	$390\ J\ kg^{-1}K^{-1}$	Specific heat capacity of copper	No
cp_p	$1200\ J\ kg^{-1}K^{-1}$	Specific heat capacity of polycarbonate	No
cp_i	$2100 + 0.6\boldsymbol{T}\ J\ kg^{-1}K^{-1}$	Specific heat capacity of ice	Yes
ρ_w	$997\ kg\ m^{-3}$	Density of water	No
ρ_{Cu}	$8960\ kg\ m^{-3}$	Density of copper	No
ρ_p	$1200\ kg\ m^{-3}$	Density of polycarbonate	No
ρ_i	$918\ kg\ m^{-3}$	Density of ice	No
L	$333.5\ kJ\ kg^{-3}$	Latent heat of water-ice transition	No
T_f	0 ℃	Temperature for water-ice phase change	No
dt	4 ℃	Temperature interval for water-ice transition	No
CR	1 ℃ min^{-1}	Cooling rate of the cold finger	No
h_c	$27.5\ W\ m^{-2}\ K^{-1}$	Heat convection coefficient	No
T_0	20 ℃	Initial temperature/ambient temperature	No

Video S1.
3D animation of the wedge design.

Video S2.
3D animation of the Bouligand-like orientation design.

Video S3.
The reconstructed μCT images of the sample fabricated in the refined mould, showing a continuous pore orientation shift in region 1-5.
